# Subpolar North Atlantic Overturning and Gyre‐Scale Circulation in the Summers of 2014 and 2016

**DOI:** 10.1029/2018JC013841

**Published:** 2018-07-02

**Authors:** N. P. Holliday, S. Bacon, S. A. Cunningham, S. F. Gary, J. Karstensen, B. A. King, F. Li, E. L. Mcdonagh

**Affiliations:** ^1^ National Oceanography Centre Southampton UK; ^2^ Scottish Association for Marine Science Oban UK; ^3^ GEOMAR Helmholtz Centre for Ocean Research Kiel Kiel Germany; ^4^ Nicholas School of the Environment Duke University Durham NC USA

**Keywords:** subpolar North Atlantic, Atlantic Meridional Overturning Circulation, isopycnal circulation, freshwater transport, heat transport

## Abstract

The Atlantic Meridional Overturning Circulation (AMOC) is a key component of the global climate system through its transport of heat and freshwater. The subpolar North Atlantic (SPNA) is a region where the AMOC is actively developed and shaped though mixing and water mass transformation and where large amounts of heat are released to the atmosphere. Two hydrographic transbasin sections in the summers of 2014 and 2016 provide highly spatially resolved views of the SPNA velocity and property fields on a line from Canada to Greenland to Scotland. Estimates of the AMOC, isopycnal (gyre‐scale) transport, and heat and freshwater transport are derived from the observations. The overturning circulation, the maximum in northward transport integrated from the surface to seafloor and computed in density space, has a high range, with 20.6 ± 4.7 Sv in June–July 2014 and 10.6 ± 4.3 Sv in May–August 2016. In contrast, the isopycnal (gyre‐scale) circulation was lowest in summer 2014: 41.3 ± 8.2 Sv compared to 58.6 ± 7.4 Sv in 2016. The heat transport (0.39 ± 0.08 PW in summer 2014, positive is northward) was highest for the section with the highest AMOC, and the freshwater transport was largest in summer 2016 when the isopycnal circulation was high (−0.25 ± 0.08 Sv). Up to 65% of the heat and freshwater transport was carried by the isopycnal circulation, with isopycnal property transport highest in the western Labrador Sea and the eastern basins (Iceland Basin to Scotland).

## Key Points

- The subpolar North Atlantic Meridional Overturning Circulation was 20.6 ± 4.7 Sv in summer 2014 and 10.6 ± 4.3 Sv in summer 2016
- The isopycnal circulation was 41.4 ± 8.2 Sv in 2014 and 58.6 ± 7.4 Sv in 2016, carrying up to 65% of the total heat and freshwater transport
- Heat transport increased with overturning circulation (maximum 0.39 PW), freshwater transport increased with isopycnal circulation (maximum ‐0.25 Sv)

## 1 Introduction

The Atlantic Meridional Overturning Circulation (AMOC) is a key component of the global climate system through its transport of heat and freshwater. The subpolar North Atlantic (SPNA) is a region where the AMOC is actively developed and shaped though mixing and water mass transformation. It is a region where large amounts of heat transported northward by the ocean are released to the atmosphere, thereby modifying the climate of northwest Europe. Changes in SPNA heat content and surface temperature are significant for many climate and weather phenomenon including rainfall in the African Sahel, Amazon, western Europe, and parts of the United States (Duchez et al., 2016; Dunstone et al., 2011; Knight et al., 2006; Sutton & Dong, 2012; Sutton & Hodson, 2005; Zhang & Delworth, 2006).

**Figure 1:**
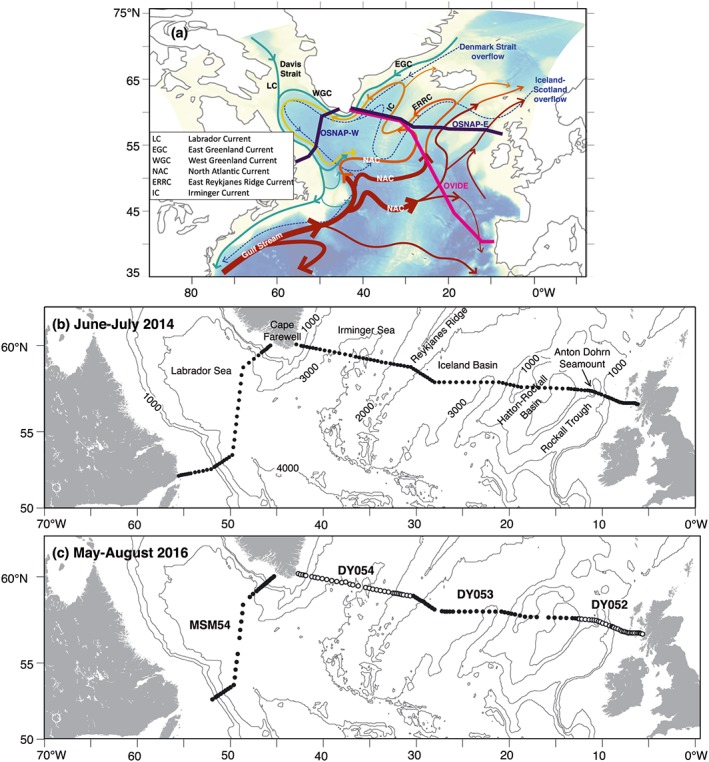
Regional circulation of the subpolar North Atlantic and location of the data used in the study. (a) Schematic circulation of the upper layer (solid arrows) and overflows (dashed arrows), superimposed by the location of the OSNAP section and array, and the OVIDE section (adapted from Daniault et al., 2016). (b) Location of conductivity‐temperature‐depth/lowered Acoustic Doppler current profiler stations taken on JR302 in June–July 2014 (OS2014); c) Location of conductivity‐temperature‐depth/lowered Acoustic Doppler current profiler stations taken in May–August 2016 on cruises MSM54, DY054, DY053, and DY052 (OS2016). See Table 1 for more information about the cruises. OSNAP = Overturning in the Subpolar North Atlantic Program; LC = Labrador Current; EGC = East Greenland Current; WGC = West Greenland Current; NAC = North Atlantic Current; ERRC = East Reykjanes Ridge Current; IC = Irminger Current.

The SPNA has complex topography with a series of basins (Figure 1) in which the large‐scale circulation is characterized by cyclonic boundary currents and interior recirculation. The North Atlantic Current (NAC) develops out of the Gulf Stream extension and turns eastward, crossing the Atlantic in a wide band between about 45°N and 55°N (Figure 1a). There are several branches of the NAC, and they flow into an eastern intergyre region in the Bay of Biscay, the Rockall Trough, the Iceland Basin, and the Irminger Sea. Part of the NAC flows into the Norwegian Sea, and some recirculates within the boundary currents of the subpolar gyre (e.g., Hansen & Østerhus, 2000).

The upper layer in the eastern basins contains a variety of Subpolar Mode Waters (SPMW) carried between fronts associated with the NAC branches (Brambilla & Talley, 2008). The Rockall Trough contains SPMW from a major southern NAC branch and also Eastern North Atlantic Water from the Biscay intergyre regions (Figure 1a); this basin contains the highest influence of subtropical water of the Overturning in the Subpolar North Atlantic Program (OSNAP) section (Holliday et al., 2015). The Iceland Basin contains two NAC branches, and in its western side there is a southward flow along the east flank of the mid‐Atlantic ridge (the East Reykjanes Ridge Current, ERRC; after Treguier et al., 2005), which is recirculating and modified NAC water (Figure 1a). The Irminger Current on the west flank of the Reykjanes Ridge is mainly recirculating ERRC that has turned north having crossed the Ridge, and is also fed in part by a minor northern branch of the NAC (e.g., Daniault et al., 2016). The various NAC branches carry the SPMW cyclonically around the area, with ongoing air‐sea interaction cooling and freshening the SPMW, especially in winter when mixing can be up to 800–1,300 m in the basins east of Greenland (e.g., Brambilla & Talley, 2008; Piron et al., 2017).

The west Irminger Sea is dominated by the southward flowing East Greenland Current (EGC, Figure 1a) that has an offshore component formed by the recirculating Irminger Current and at the shelf break a component that flows south through the Denmark Strait (Sutherland & Pickart, 2008). The EGC follows the bathymetry around Cape Farewell (Holliday et al., 2009) and becomes the West Greenland Current (WGC), which overall traces a path around the rim of the Labrador Sea at the continental shelf break (Figure 1a), eventually becoming part of the outer, largely barotropic component of the Labrador Current in the western Labrador Sea (Hall et al., 2013). Within the center of the Irminger and Labrador Seas, away from the relatively saline boundary currents, the upper layers contains fresh, stratified sub‐Arctic surface water. This water type becomes subducted as Subarctic Intermediate Water (SAIW) within the NAC zone and forms part of the deeper, permanent thermocline of the basins east of the Reykjanes Ridge (Arhan, 1990; Harvey, 1982).

Around the rims of the western SPNA, two shallow, fresh and buoyant currents advect cold water southward from the Arctic and Nordic Seas. In the Irminger Sea the East Greenland Coastal Current (EGCC) flows southward to Cape Farewell (Bacon et al., 2002; Sutherland & Pickart, 2008), follows the topography around Cape Farewell, after which it becomes known as the West Greenland Coastal Current. On the Labrador and Newfoundland coasts the Labrador Current has a cold, fresh baroclinic component sitting over the shelf break (Lazier & Wright, 1993).

The intermediate layer of the SPNA is filled with Labrador Sea Water (LSW), formed mainly in the Labrador Sea and also in the Irminger Sea, from where it spreads into the eastern basins and becomes warmer and saltier through mixing with surrounding water masses (Kieke & Yashayaev, 2015; Yashayaev et al., 2007). The interior Labrador and Irminger Seas both contain recirculation features especially evident at middepths (Lavender et al., 2005). At depth sit the dense northern overflow waters, the Iceland‐Scotland Overflow Water (ISOW) that enters the subpolar basins in the east, and the Denmark Strait Overflow Water (DSOW) in the west (Dickson & Brown, 1994). Both overflow water types flow cyclonically in deep western boundary currents (DWBCs; Figure 1a) and are continuously modified by mixing before they leave the region.

In its simplest form, the concept of the AMOC is a northward flow of warm salty water in the upper layers of the ocean balanced by a return flow of denser cold, fresh water in intermediate and deep layers, with much of this transformation of surface to deep water taking place in the SPNA and the Nordic Seas. In the subtropical Atlantic Ocean the AMOC is commonly defined as the total northward transport of the zonally integrated meridional flow (usually the maximum of the overturning stream function, AMOC_*z*_), where the subscript *z* indicates that the zonal integral is taken in depth space (e.g., McCarthy et al., 2015). In the subpolar North Atlantic, the prevalence of diapycnal mixing and the region‐wide sloping of isopycnals means that the subpolar AMOC is more appropriately considered in density coordinates (AMOC_*σ*_; Li et al., 2017; Mercier et al., 2015; Xu et al., 2016). The residuals from the mean transport profile in density coordinates describe the gyre‐scale or isopycnal circulation (Mercier et al., 2015).

A recent international observational program, OSNAP, was designed to study the subpolar AMOC and gyre circulation (http://www.o-snap.org; Li et al., 2017; Lozier et al., 2017). The OSNAP array was deployed in the summer of 2014 for the purpose of recording continuous transbasin observations of volume, heat, and freshwater transport in the region. The array uses moored instruments, gliders, and floats (RAFOS and Argo) to measure velocity, temperature, and salinity along a section from Canada to Greenland to Scotland. The moorings are located in the boundary currents of the four major basins of the subpolar region (the Labrador Sea, Irminger Sea, Iceland Basin, and Rockall Trough, Figure 1), and the gliders and floats provide additional information in the regions between. The array will provide monthly estimates of the overturning circulation, heat, and freshwater transport, along with the velocity field at low spatial resolution (see Lozier et al., 2017, for more details).

The OSNAP array builds on the knowledge gained from previous and ongoing SPNA measurement programs, including the following. The 53°N Labrador Sea moored array forms the western end of the OSNAP array and measures the DWBC (Zantopp et al., 2017). The Extended Ellett Line annual repeat hydrography program occupied since 1975 forms the eastern end of the OSNAP array in the Rockall Trough (Figure 1; Holliday et al., 2015). The OVIDE biennial repeat hydrography program observes the Meridional Overturning Circulation (MOC) in the eastern SPNA (Figure 1a; Daniault et al., 2016; Mercier et al., 2015). The AR7W section in the Labrador Sea is an annual repeat hydrography section that lies just to the north of the OSNAP line (Hall et al., 2013; Yashayaev & Loder, 2016). The AR7E/60°N repeat hydrography program east of Greenland has provided estimates of mean MOC and heat flux in the summer of the 2000s (Sarafanov et al., 2012). Ship‐of‐opportunity measurement of upper ocean currents and surface properties at ~60°N has been analyzed for more recent estimates of MOC and property fluxes east of Greenland (Rossby et al., 2017). The OSNAP array also enhances measurements made by moored arrays at the Greenland to Scotland sill (Hansen et al., 2017; Harden et al., 2016) and by high precision pressure sensors at 47°N (Roessler et al., 2015). Uniquely, the OSNAP array measures the circulation over the full depth and full width of the SPNA, including Labrador Sea and east of Greenland, on monthly timescales.

In this study we present detailed views of the full‐depth temperature, salinity, density, and velocity fields from high spatial resolution hydrographic sections along the OSNAP line taken at the start of the program in June–July 2014 and during mooring turnaround cruises in May–August 2016 (Figure 1). These sections provide detailed, fine structure observations of temperature, salinity, and velocity that will provide independent calibration points for the OSNAP array, which is more limited spatially and vertically. No previous study has presented estimates of circulation and volume and property transport from a section that is well resolved spatially and covers both the Labrador Sea and the eastern SPNA. Here we derive estimates of the Atlantic Meridional Overturning Circulation (AMOC_*σ*_), the isopycnal (gyre‐scale) circulation, and their components of net heat and freshwater transport and identify the key parts of the section for heat and freshwater transport. We describe the character of the SPNA AMOC, which is complicated by the presence of the cold and fresh shallow boundary currents (LC, EGC, EGCC, WGC, and West Greenland Coastal Current). We examine the consistency and differences between the two sections, and finally, we discuss our results in the context of existing estimates.

## 2 Data

Details of the cruise data used in this analysis are given in Table 1. The uncertainty from using a collection of cruises to construct the 2016 section is discussed in section 7. We refer to the two OSNAP sections as OS2014 and OS2016 to emphasize that the transport estimates and properties refer to the period of time during which the sections were completed. Stations were occupied with horizontal resolution of 30 km or less (closer over rapidly changing bathymetry; Figure 1), with a full suite of conductivity‐temperature‐depth (CTD) sensor measurements (pressure, temperature, conductivity, and dissolved oxygen concentration) and water samples for conductivity calibration (Figure 1). CTD data were calibrated with Standard Sea Water samples and laboratory calibrations to GO‐SHIP standards (salinity 0.002, pressure 1 dbar, and temperature 0.002 °C, http://www.go-ship.org). See Table 1 for cruise reports.

**Table 1:** Details of Cruise Data Sets Used in the Analysis

Cruise	Dates	Principal scientist	Location	Number of stations	Cruise report and data
OS2014					
JR302	6 Jun to 21 Jul	B. King and N. P. Holliday, UK	Canada to Greenland to Scotland	146	http://www.bodc.ac.uk/resources/inventories/cruise_inventory/report/15037
OS2016					
MSM54	13 May to 7 Jun	J. Karstensen, Germany	Labrador Sea	38	http://www.pangaea.de/expeditions/cr.php/Merian
DY054	27 Jul to 17 Aug	N. P. Holliday, UK	Irminger Sea	34	http://www.bodc.ac.uk/resources/inventories/cruise_inventory/report/16034
DY053	29 Jun to 23 Jul	S. Cunningham, UK	Iceland Basin and Hatton‐Rockall Basin	38	http://www.bodc.ac.uk/resources/inventories/cruise_inventory/report/16033
DY052	7 to 24 June	S. Gary, UK	Rockall Trough	34	http://www.bodc.ac.uk/resources/inventories/cruise_inventory/report/16032

*Note*. The number of stations refers to those on the OSNAP section that were used in this analysis; more stations were taken on each cruise. See Figure 1 for station positions.

Lowered (L‐) Acoustic Doppler current profilers (LADCPs) measured full‐depth currents at each cast except for a small number of very shallow stations. LADCP data on the UK cruises (JR302, DY052, and DY054; Table 1) were processed using the Lamont Doherty Earth Observatory IX software v8 (http://www.ldeo.columbia.edu/~ant/LADCP) and the GEOMAR LADCP processing software V10.12 on the German cruise (MSM54; Table 1). LADCP absolute velocities from these processing methods have an estimated uncertainty of 0.02–0.03 m/s (Holliday et al., 2009; Thurnherr, 2010). The presence of high numbers of scatterers throughout the water column means that good velocity data were returned at all depths. Shipboard (S‐) ADCP (SADCP) data on UK cruises were processed using the University of Hawaii's Common Ocean Data Access System, using the heading information from the ship's Global Positioning System data stream and calibrated transducer heading misalignment (for more details see King & Holliday, 2015). The barotropic tides at the time of each cast were obtained from the Oregon State University Tidal Prediction software (http://volkov.oce.orst.edu/tides/otps.html), and once de‐tided, the *u* and *v* components were rotated to provide the velocity normal to the section, *v*_LADCP_ (positive values to the north of the section).

CTD and LADCP data were interpolated onto a vertical grid with 20‐dbar intervals. For velocity, transport, and flux calculations we retain the original horizontal station spacing. For examining the difference in properties between the two sections, we interpolated the data to a horizontal grid with 10‐km spacing. Salinity is reported on the practical salinity scale, and potential density is referenced to the surface.

## 3 Methods

### 3.1 Derivation of the Total Velocity Field

Vertical geostrophic shear is derived from the density gradient between CTD stations, and further sources of information are needed to obtain the total, absolute velocity field. We compute geostrophic shear from the density field, add an observed reference velocity, add Ekman velocity computed from wind stress, and then apply an adjustment to meet specified volume transport constraints.

We derive the initial observed cross‐section velocity field *v*_obs_ as follows:

(1) $$v_{\mathrm{obs}}\left(x,z\right)=v_{g}\left(x,z\right)+v_{\mathrm{ref}}\left(x\right)+v_{\mathrm{ek}}\left(x,z\right)$$

where *v*_*g*_ is geostrophic velocity, *v*_ref_, is reference velocity, *v*_ek_ is Ekman velocity, *x* is the along‐track direction, and *z* is depth. *v*_*g*_ is computed from the temperature and salinity profile for each station pair, with an initial level of no motion at the seafloor (giving one profile per station pair). We obtain the reference velocity from LADCP data (*v*_ref_) and given by the following:

(2) $$v_{\mathrm{ref}}\left(x\right)=\bar{v_{\text{ladcp}}\left(x,z\right)-v_{g}\left(x,z\right)}$$

where *v*_ladcp_ is the cross‐track component of the station pair mean LADCP velocity profile (i.e., the average of the two de‐tided casts). The overbar represents the average over all depths below 250 m (in order to exclude surface motions, which are dominated by ageostrophic transient currents). SADCP data are used for a small number of shallow stations with no LADCP data. Acoustic Doppler current profiler (ADCP)‐derived reference velocities are particularly valuable in the DWBCs of the Labrador and Irminger Seas and in the Iceland Basin where there is strong vertical shear. Additionally, they provide high horizontal resolution in narrow boundary currents, which can be underestimated and underresolved by altimeter‐derived surface reference velocities (e.g., Gourcuff et al., 2011; Sherwin et al., 2015). In the Labrador Sea and Irminger Seas the LADCP reference value adds up to 5 Sv to the transport within the DWBCs over that estimated when using reference velocities from altimeter‐derived surface geostrophic velocity (the latter reported for JR302 [Table 1] in Johnson et al., 2015).

In the bottom triangles (the area of water below the deepest common level of a station pair where we have neither station pair mean LADCP nor geostrophic velocity) we assume a constant velocity equal to that at the deepest common level (after Holliday et al., 2009). The transport in the bottom triangles accounts for 1.1 Sv accumulated along the OS2014 section and −0.17 Sv accumulated along the OS2016 section.

Wind data for the time period of the cruise were obtained from European Centre for Medium‐Range Weather Forecasts (http://apps.ecmwf.int/datasets/data/interim-full-daily/). Ekman transport was then computed from ERA Interim winds, following the method described in McCarthy et al. (2015). Zonal and meridional 10‐m wind data at grid points matching the cruise track were extracted and rotated to compute cross‐track wind stress and Ekman transport. We use the reanalysis product rather than the in situ wind data because the ship measurements are affected by airflow distortion. Ekman velocity is added to the top 55 m and is obtained by dividing the transport by the cross‐sectional area (distance × depth). The net Ekman transport is near zero at this latitude: 0.04 Sv integrated across OS2014 and 0.02 Sv integrated across OS2016.

The volume transport normal to the section was computed from the velocity field and cross‐sectional area (*A*, m^2^) as follows:

(3) $$T_{\mathrm{obs}}=\sum_{x_{w}}^{x_{e}}\sum_{z_{\max}}^{z_{\min}}v_{\mathrm{obs}}\left(x,z\right)\cdot A\left(x,z\right)\;$$

and is reported in units of Sv (1 Sv = 10^6^ m^3^ s^−1^). We derive total volume transport, *T*_total_ (and therefore total velocity, *v*_total_), by adding a uniformly distributed adjustment (*T*_adj_) to meet volume transport constraints from the literature.

(4) $$T_{\text{total}}=T_{\mathrm{obs}}+T_{\mathrm{adj}},$$

(5) $$v_{\text{total}}\left(x,z\right)=v_{\mathrm{obs}}\left(x,z\right)+v_{\mathrm{adj}}\left(x,z\right).$$

Long‐term observations show that there is a mean throughflow of 0.8 ± 0.1 Sv through the Bering Straits into the Arctic Ocean (Woodgate & Aagaard, 2005). Since the Arctic basin is open only to the Pacific through the Bering Strait and to the SPNA through a series of openings to the north of the OSNAP section, to conserve mass between the Bering Strait and OSNAP section, there must also be a mean throughflow of order −0.8 Sv across the OSNAP section, that is, *T*_total_ = −0.8 Sv. We refine this geographically: Long‐term measurements though the Davis Strait into the Labrador Sea have a mean transport of −1.6 ± 0.5 Sv (Curry et al., 2014), and the OVIDE program estimates a long‐term mean of 1.0 ± 0.9 Sv between Greenland and Portugal (Mercier et al., 2015). We compute *v*_adj_ separately for OSNAP‐W and OSNAP‐E to satisfy our constraints of *T*_total_ = −0.8 Sv: *T*_total (OSNAP‐W)_ = −1.6 Sv and *T*_total (OSNAP‐E)_ = 0.8 Sv. The adjustment velocities are applied uniformly across each subsection: −0.002 m/s for OSNAP‐W and −0.003 m/s for OSNAP‐E in OS2014 and 0.007 m/s for OSNAP‐W and 0.001 m/s for OSNAP‐E in OS2016. The final velocity field *v*_total_ is subsequently used for all the volume and property transport estimates as we describe next.

### 3.2 Overturning Circulation, Throughflow, and Isopycnal Transport

**Figure 2:**
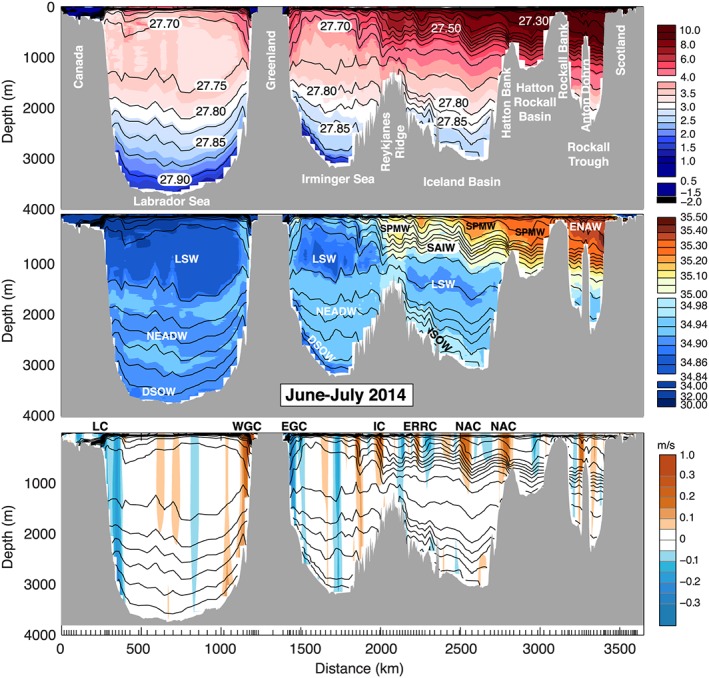
The June–July 2014 (OS2014) section. Potential temperature °C (top panel), salinity (middle panel), and velocity orthogonal to the section (bottom panel, positive is to the north of the section). Black lines are contours of potential density (sigma0) at intervals of 0.050 for <27.800 kg/m^3^ and intervals of 0.025 for denser water. Major water masses are labeled: Labrador Sea Water (LSW), North East Atlantic Deep Water (NEADW), Denmark Strait Overflow Water (DSOW), Subpolar Mode Water (SPMW), Subarctic Intermediate Water (SAIW), Iceland‐Scotland Overflow Water (ISOW), and Eastern North Atlantic Water (ENAW). Major current systems are labeled: Labrador Current (LC), West Greenland Current (WGC), East Greenland Current (EGC, including the East Greenland Coastal Current), Irminger current (IC), East Reykjavik Ridge Current (ERRC), North Atlantic Current (NAC), and Atlantic Current (AC).

**Figure 3:**
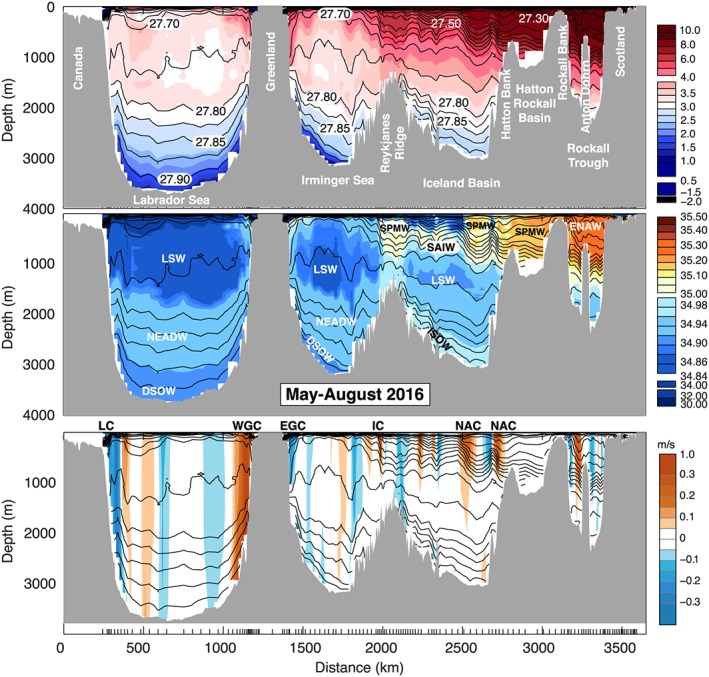
The May–August 2016 (OS2016) section. Potential temperature °C (top panel), salinity (middle panel), and velocity orthogonal to the section (bottom panel, positive is to the north of the section). Black lines are contours of potential density (sigma0) at intervals of 0.050 for <27.800 kg/m^3^ and intervals of 0.025 for denser water. Major water masses and current systems are labeled as for Figure 2.

The isopycnals of the SPNA slope down from west to east (Figures 2 and 3), and their gradients change across individual basins and with depth. Any chosen depth range on the OSNAP section thus contains water masses with a wide range of densities and will include currents flowing in different directions that are not part of the same recirculation features. For this reason we compute transports and circulation metrics in density coordinates, thereby more appropriately describing the subpolar circulation (Mercier et al., 2015; Xu et al., 2016). We regrid our velocity (*v*_total_) and property fields (*θ, S*) from depth (*z*) to potential density (*σ*) at a resolution of 0.01 kg/m^2^.

According to Bryden and Imawaki (2001) and adapted by Mercier et al. (2015), the volume transport across a (near) zonal coast‐to‐coast section can be decomposed into the net throughflow (the barotropic component, $\bar{v}$), a closed vertical cell (the zonally averaged meridional component, ⟨*v*⟩), and a closed *horizontal* or isopycnal circulation cell (the deviations from the zonal average, *v*′), where

(6) $$v_{\text{total}}\left(x,\sigma \right)=\bar{v}+\left\langle v\right\rangle \left(\sigma \right)+v\prime \left(x,\sigma \right).$$

Similarly, the potential temperature and salinity fields can be decomposed into components associated with the throughflow, the meridional overturning (diapycnal) circulation and the gyre‐scale (isopycnal) circulation:

(7) $$\theta \left(x,\sigma \right)=\bar{\theta }+\left\langle \theta \right\rangle \left(\sigma \right)+\theta^{\prime }\left(x,\sigma \right).$$

(8) $$S\left(x,\sigma \right)=\bar{S}+\left\langle S\right\rangle \left(\sigma \right)+S^{\prime }\left(x,\sigma \right).$$

The volume transport profiles associated with the meridional overturning (*T*_amoc_) and isopycnal circulation (*T*_gyre_) are defined as

(9) $$T_{\text{amoc}}\left(\sigma \right)=\sum_{x_{w}}^{x_{e}}A\left(x,\sigma \right)\cdot \left\langle v\right\rangle \left(\sigma \right)\;$$

(10) $$T_{\text{gyre}}\left(x\right)=\sum_{\sigma_{\max}}^{\sigma_{\min}}A\left(x,\sigma \right)\cdot v^{\prime }\left(x,\sigma \right)\;$$

In section 1 we note that the concept of the AMOC with a northward flowing upper limb and a southward flowing deeper limb is prevalent but that the complexity of the circulation in the SPNA means that the AMOC has at least two potential definitions, resulting in two views of its mean and variability. We present two definitions of AMOC_*σ*_ which we discuss later; the first is the maximum value of the overturning stream function (*T*_amoc_ accumulated from low to high density, AMOC_*σ*‐max_ adapted from Mercier et al., 2015) and the second is the sum of all the northward transport in the upper layer (lighter than density at the maximum value of the overturning stream function) of *T*_amoc_ (AMOC_*σ*‐*n*_ adapted from Li et al., 2017). The maximum value of *T*_gyre_ accumulated from west to east gives a section estimate of the isopycnal transport.

### 3.3 Heat and Freshwater Transport

The section temperature transport (HT) and heat transport associated with the closed overturning (HT_amoc_) and isopycnal (HT_gyre_) circulation cells are defined as follows and given in units of petawatts:

(11) $$\mathrm{HT}=\iint \rho C_{p}v_{\text{total}}\theta \;dxd\sigma \;$$

(12) $${\mathrm{HT}}_{\mathrm{moc}}=\iint \rho C_{p}\left\langle v\right\rangle \left\langle \theta \right\rangle \;dxd\sigma \;$$

(13) $${\mathrm{HT}}_{\text{gyre}}=\iint \rho C_{p}v\prime \theta \prime \;dxd\sigma \;$$

where *ρ* is seawater density and *C*_*p*_ is specific heat capacity of seawater.

Rather than simply computing the salt transport at the OSNAP section, we want to use the salinity and velocity information to quantify the more climate‐relevant freshwater transport. That is usefully approached by considering a closed ocean basin (the wider Arctic, bounded by the Bering Strait and the OSNAP section) as described by Bacon et al. (2015). Large amounts of freshwater are added to the ocean, while salt is conserved in this bounded Arctic region; this, along with mixing and cooling, is the process by which the warm, saline, northbound surface waters are transformed into colder and fresher returning layers. The boundary approach allows us to compute the freshwater added to the ocean between the Bering Strait and the OSNAP section without invoking a reference salinity (which is subjective) and without needing to know the throughflow transport (which we have set to historical values). The mathematical derivation of the approach is explained and tested in Bacon et al. (2015), and freshwater flux though the boundary (*F*_*A*_) is defined as

(14) $$F_{A}=-\iint \frac{\left\{S_{A}\right\}\left\{v_{A}\right\}}{\bar{S_{A}}}dxd\sigma$$

where subscript *A* indicates the extended Arctic boundary consisting of the OSNAP section and the Bering Strait, overbar indicates the boundary area mean, and curly brackets indicate anomalies with respect to the mean. *F*_*A*_ is the equivalent of the freshwater divergence described by McDonagh et al. (2015).

We use climatological means for the Bering Strait (transport 0.8 Sv and salinity 32.50; Woodgate et al., 2005, 2006), together with the measured salinity and velocity from the OSNAP section to construct the Arctic boundary velocity and salinity fields (34.269 for OS2014 and 34.876 for OS2016). The freshwater transport at the OSNAP section (FT) is *F*_*A*_ minus the freshwater transport at the Bering Strait.

The freshwater fluxes associated with the overturning circulation, FT_amoc_, and isopycnal circulation, FT_gyre_, at the OSNAP section are defined as follows:

(15) $${\text{FT}}_{\text{amoc}}=-\int \frac{\left\langle S\right\rangle -\bar{S_{A}}}{\bar{S_{A}}}\left\langle v\right\rangle d\sigma$$

(16) $${\text{FT}}_{\text{gyre}}=-\iint \frac{S^{\prime }-\bar{S_{A}}}{\bar{S_{A}}}v\prime dxd\sigma .$$

### 3.4 Uncertainty Estimates

**Figure 4:**
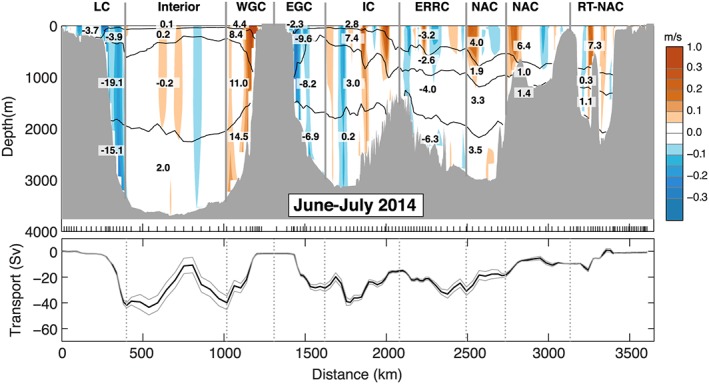
The June–July 2014 (OS2014) section velocity (m/s) and transport (Sv). Top panel is velocity orthogonal to the section (as shown in Figure 2), overlaid with volume transport in segments separated geographically (vertical lines) and by isopycnals 27.50, 27.70, and 27.80 kg/m^3^ (black lines, see Figure 2). See Table 2 for uncertainty estimates. Bottom panel is top‐to‐bottom accumulated transport (west to east). Positive is to the north of the section; uncertainties are estimated from lowered Acoustic Doppler current profiler measurements. Major current systems are labeled as for Figure 2. RT is Rockall Trough.

**Figure 5:**
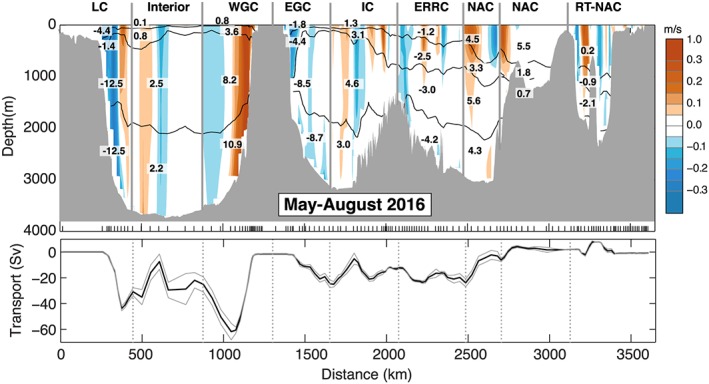
The May–August 2016 (OS2016) section velocity (m/s) and transport (Sv). Top panel is velocity orthogonal to the section (as shown in Figure 3), overlaid with volume transport in segments separated geographically (vertical lines) and by isopycnals 27.50, 27.70, and 27.80 kg/m^3^ (black lines; see Figure 3). See Table 2 for uncertainty estimates. Bottom panel is top‐to‐bottom accumulated transport (west to east). Positive is to the north of the section; uncertainties are estimated from lowered Acoustic Doppler current profiler measurements. Major current systems are labeled as for Figure 2. RT is Rockall Trough.

For estimating the uncertainty in top‐to‐bottom transport we combine errors from sources assumed to be independent: the LADCP measurements, the mass balance constraints (Bering Strait and Davis Strait), the presence of internal waves causing isopycnal heave, and bottom triangles. For the mass constraint uncertainty we use 2 standard deviations of the long‐term measurements: 0.2 Sv at Bering Strait (Woodgate & Aagaard, 2005) and 1.0 Sv at Davis Strait (Curry et al., 2014). The combined instrument and processing uncertainty from each individual LADCP velocity profile is estimated as 0.02 m/s (Hall et al., 2013; Holliday et al., 2009; Thurnherr, 2010), and taking this to be consistent in the vertical and random, we compute an uncertainty from the reference velocity for each part of the section (Figures 4 and 5 and Table 2). For the top‐to‐bottom transport, the reference velocity uncertainty is equivalent to 12.0 Sv for OS2014 (section area 7.2 × 10^9^ m^2^, number of stations 145) and 11.4 Sv for OS2016 (section area 6.8 × 10^9^ m^2^, number of stations 144). Bottom triangle errors are estimated at 0.03 m/s (after Holliday et al., 2009), giving a small additional uncertainty of 0.3 Sv for both sections. Ganachaud (2003) estimated that uncertainty from isopycnal heave as a result of the presence of internal waves could add an uncertainty of ±3.3 Sv to a section and we adopt that estimate here. Together, these give an RMS (root‐mean‐square) uncertainty of 12.4 Sv in the top‐to‐bottom transport in OS2014 and 11.9 Sv in OS2016.

**Table 2:** Volume Transport in Hydrographic Features and Layers (Units Are Sv)

Feature	Year	Upper Ocean (<27.50 kg/m^3^)	Thermocline and SAIW (27.50–27.70 kg/m^3^)	Labrador Sea water (27.70–27.80 kg/m^3^)	Overflow layer (>27.80 kg/m^3^)	Full depth
Labrador Current	2014	−3.7 ± 0.3	−3.9 ± 0.2	−19.1 ± 0.8	−15.1 ± 0.5	−41.9 ± 1.8
	2016	−4.4 ± 0.1	−1.4 ± 0.2	−12.5 ± 1.3	−12.5 ± 1.0	−30.8 ± 2.5
Labrador Sea Interior	2014	0.1 ± 0.1	0.2 ± 1.0	−0.2 ± 6.2	2.0 ± 5.6	1.9 ± 13.0
	2016	0.1 ± 0.1	0.8 ± 0.7	2.5 ± 5.8	2.2 ± 5.4	5.7 ± 12.0
West Greenland Current	2014	4.4 ± 0.1	8.4 ± 0.4	11.0 ± 1.3	14.5 ± 1.0	38.4 ± 2.8
	2016	0.8 ± 0.0	3.6 ± 0.2	8.2 ± 2.3	10.9 ± 1.6	23.5 ± 4.3
East Greenland Current	2014	−2.3 ± 0.1	−9.6 ± 0.4	−8.2 ± 1.2	−6.9 ± 0.9	−27.0 ± 2.7
	2016	−1.8 ± 0.1	−4.4 ± 0.2	−8.5 ± 1.8	−8.7 ± 1.1	−23.5 ± 3.2
Irminger Current	2014	2.8 ± 0.3	7.4 ± 1.0	3.0 ± 2.3	0.2 ± 1.9	13.6 ± 5.5
	2016	1.3 ± 0.1	3.1 ± 0.5	4.6 ± 2.5	3.0 ± 1.3	12.0 ± 4.5
West Iceland Basin	2014	−3.2 ± 0.9	−2.6 ± 0.9	−4.0 ± 1.7	−6.3 ± 1.3	−16.0 ± 4.8
(East Reykjanes Ridge Current	2016	−1.1 ± 0.6	−2.5 ± 1.0	−3.0 ± 1.7	−4.2 ± 1.0	−10.8 ± 4.3
Central Iceland Basin	2014	4.0 ± 1.1	1.9 ± 0.8	3.3 ± 1.8	3.4 ± 1.4	12.7 ± 5.2
(North Atlantic Current)	2016	4.5 ± 1.0	3.3 ± 0.8	5.6 ± 1.8	4.3 ± 1.4	17.8 ± 5.1
East Iceland Basin	2014	6.4 ± 1.4	1.0 ± 0.5	1.4 ± 0.1	not present	8.7 ± 2.1
(North Atlantic Current)	2016	5.5 ± 1.4	1.8 ± 0.3	0.7 ± 0.2	negligible	8.1 ± 2.0
Rockall Trough	2014	7.3 ± 0.8	0.3 ± 0.2	1.1 ± 0.3	0.1 ± 0.0	8.7 ± 1.4
(North Atlantic Current)	2016	0.2 ± 0.7	−0.9 ± 0.2	−2.1 ± 0.3	negligible	−2.8 ± 1.2

*Note*. Density ranges are the following: upper ocean <27.50 kg/m^3^; thermocline and Subarctic Intermediate Water 27.50–27.70 kg/m^3^; Labrador Sea Water 27.70–27.80 kg/m^3^; overflows, including Iceland‐Scotland Overflow Water and Denmark Strait Overflow Water, >27.80 kg/m^3^. Northward transports are positive and error bars give uncertainty (see section 3).

For the AMOC we compute the RMS uncertainty in the layer lighter than the maximum of the overturning stream function, giving 4.7 Sv for OS2014 and 4.3 Sv for OS2016. For isopycnal circulation uncertainty we compute the RMS uncertainty for top‐to‐bottom transport in the eastern gyre area between Scotland and the location of the maximum of the isopycnal circulation in the Irminger Sea, giving 8.2 Sv for OS2014 and 7.4 Sv for OS2016. Since volume transport is the most important factor in determining the property fluxes (e.g., Rossby et al., 2017), the heat and freshwater flux uncertainties are estimated as proportional to the volume transport uncertainty.

## 4 Properties and Circulation in Summer 2014 and Summer 2016

We first describe the properties, circulation, and transport observed in the two sections, highlighting the consistencies and differences between the two occupations. We approach this by dividing the sections geographically and quasi‐vertically into major currents, water masses, and basins (Figures 2, 3, 4, 5 and Table 2). We divide the water column into four main density layers: the upper ocean (<27.50 kg/m^3^), which includes a shallow seasonally stratified layer; a shallow to middepth layer (27.50–27.70 kg/m^3^); the LSW layer (27.70–27.80 kg/m^3^); and the overflow layer (>27.80 kg/m^3^). We delineate the major currents geographically by choosing a location nearest to a zero isotach (Figures 4 and 5). The estimated transport in currents adjacent to major recirculation features or eddies can be sensitive to this location, and we highlight the cases where the apparent synoptic transport may be affected by recirculation or an eddy. In the following text and figures the sign convention for velocity and transport is such that the positive direction is always toward the north of the section.

### 4.1 Rockall Trough

**Figure 6:**
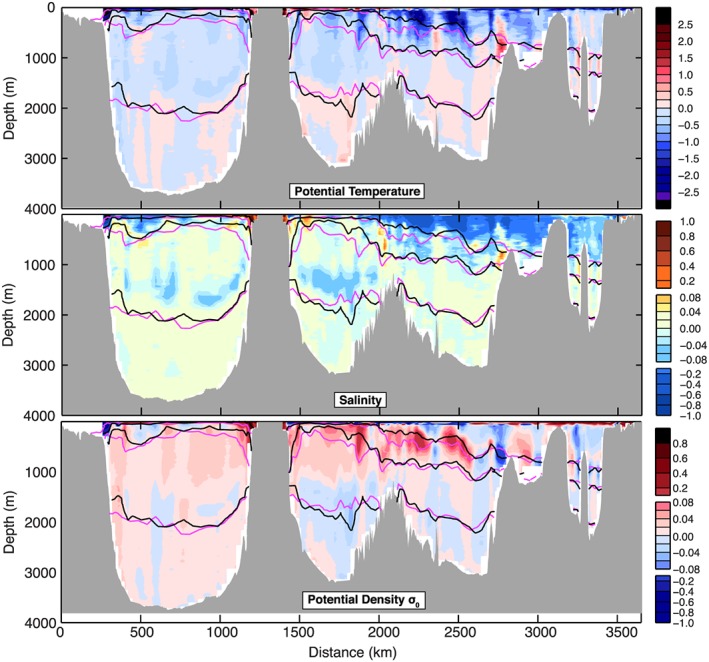
Property differences between the two sections (OS2016 minus OS2014). Top panel is potential temperature (°C), middle panel is salinity, and bottom panel is potential density (kg/m^3^). Isopycnals 27.50, 27.70, and 27.80 kg/m^3^ overlain in magenta (OS2014) and black (OS2016).

This easternmost basin contains the warmest (>9.0 °C) and most saline (>35.20) upper ocean and thermocline waters (Figures 2 and 3). In both sections a strong northward jet west of midbasin Anton Dohrn seamount is observed in the middepth and upper layer (<27.70 kg/m^3^), but the presence of a southward flow east of the seamount in 2016 means that the net transport of upper ocean and thermocline has a very high range, with 7.6 ± 1.0 Sv in OS2014 and −0.7 ± 0.9 Sv in OS2016 (Figures 4 and 5 and Table 2). There is a core of high salinity water adjacent to the continental shelf break, which is usually associated with a shelf‐edge current (Holliday et al., 2015), but neither section has a clear northward current there. Below the seasonally stratified layer, the upper 1,000 m of the Rockall Trough is cooler, fresher, and less dense in OS2016 (Figure 6).

The intermediate and deepest layers of the Rockall Trough contain modified LSW (Holliday et al., 2000) with low velocity and a small net transport (1.1 ± 0.3 Sv in OS2014 and − 2.1 ± 0.3 Sv in OS2016).

### 4.2 Iceland Basin and Hatton‐Rockall Basin

There is notable eddy activity in the Iceland Basin, but the two NAC jets are observed in consistent locations and with similar transports in both years (Figures 2 and 3). The NAC jet in the central Iceland Basin carried 5.9 ± 1.9 Sv in OS2014 and 7.8 ± 1.7 Sv in OS2016, and the jet in the east Iceland Basin (on the flank of the Hatton Bank) transported 7.4 ± 1.9 Sv in OS2014 and 7.3 ± 1.7 Sv in OS2016 (Figures 4 and 5). Our transport totals for the east jet include a small amount of recirculation within the shallow Hatton‐Rockall Basin where velocities are very low.

Our estimates of the transport of the ERRC are more variable than the NAC jets because of transport introduced by eddies and the sensitivity of the estimates to the location of the boundary. In OS2014 the net transport of the upper ocean and thermocline within the ERRC and eddies west of the central NAC jet was estimated at −5.8 ± 1.8 Sv, and in OS2016 it was −3.6 ± 1.6 Sv (Figures 4 and 5).

The upper ocean and thermocline of the Iceland Basin show a notable cooling and freshening between OS2014 and OS2016, with the largest changes and greatest increase in density associated with the three major currents in the upper 500 m (the ERRC and two NAC jets; Figure 6). The cooling and freshening extends into the thermocline layer, which contains SPMW carried by the NAC currents, and SAIW that has origins in the central subpolar gyre. Properties below the thermocline did not change much between the two sections.

In the LSW layer the Iceland Basin has low velocities in both sections, with broadly cyclonic flow (Figures 4 and 5). In OS2014 the net transport through the Iceland Basin below the permanent thermocline was estimated at −2.2 ± 6.3 Sv with −2.9 Sv in the overflow layer, and in OS2016 it was estimated at 3.3 ± 6.3 Sv with 0.1 Sv in the overflow layer.

### 4.3 Irminger Sea

In contrast with the Iceland Basin, the velocity field in the Irminger Sea shows circulation features that are deep reaching (surface to seafloor), though velocities decrease in magnitude in the intermediate layer, and in the deepest layers in the east of the basin (Figures 2 and 3).

We estimate the Irminger Current system (the main current plus the associated eddies and including the upper ocean and thermocline) as transporting 10.2 ± 1.3 Sv in OS2014 and 4.4 ± 0.6 Sv in OS2016 (Figures 4 and 5). Interestingly though, unlike the NAC branches in the Iceland Basin, the properties of the Irminger Current are relatively unchanged between OS2014 and OS2016, except in the seasonally stratified layer (here <27.50 kg/m^3^; Figure 6).

The intermediate layer of the Irminger Sea is filled with LSW and consistent with evidence for local deep convection into the LSW density range in the winter of 2014/2015 (de Jong & de Steur, 2016; Piron et al., 2017). The LSW was 0.5 °C cooler in OS2016, though only the LSW below 1,000 m was notably fresher (−0.04; Figure 6). The net transport within the LSW layer in the Irminger Sea as a whole was −5.1 ± 3.5 Sv in OS2014 and −3.9 Sv ± 4.2 Sv in OS2016 (Figures 4 and 5). The net transport though the basin in waters in the overflow layer (denser than 27.80 kg/m^3^) was −6.7 ± 2.8 Sv in OS2014 and − 5.7 ± 2.4 Sv in OS2016 (Figures 4 and 5).

The western boundary current, formed of the EGCC and the EGC, is deep reaching, but in both sections there is evidence of reduced velocity around ~2,000 m (the base of the LSW; Figures 4 and 5). Above the LSW, the transport within the EGC/EGCC was −11.1 ± 0.5 Sv in OS2014 and − 6.2 ± 0.3 Sv in OS2016. For the full‐depth western boundary current the transport was −27.0 ± 2.7 Sv in OS2014 and −23.5 ± 4.3 Sv in OS2016. Note that these estimates are sensitive to the location of the boundary between the EGC and Irminger Current and the uncertainty estimates indicate no significant change observed from OS2014 to OS2016. In contrast to the NAC water in the Iceland Basin, the EGC/EGCC waters were warmer (+2.0 °C) and more saline (+0.6) in OS2016.

### 4.4 Labrador Sea

The Labrador Sea is dominated by the fast, deep reaching boundary currents: the WGC and Labrador Current systems and a strong midbasin recirculation feature. In OS2014 the WGC system had a top‐to‐bottom transport of 38.4 ± 2.8 Sv (Figure 4), while in OS2016 it was estimated as much less (23.5 ± 4.3 Sv) because it includes large recirculation (strong southward velocity adjacent to the northward current, Figure 5). The full‐depth boundary current in the western Labrador Sea transported −41.9 ± 1.8 Sv in OS2014 and −30.8 ± 2.5 Sv in OS2016, with uncertainty in all of these estimates introduced by the interior recirculation and the lack of clarity over the lateral extent of the boundary currents.

Similar to the upstream EGC/EGCC, the shallow part of the WGC (shelf and shelf break) was warmer (+2.0 °C), more saline (+0.6), and more dense in OS2016 (Figure 6). In contrast, the shallow Labrador Current on west side of the basin was lighter, fresher, and colder in OS2016 (Figure 6). Below the seasonally stratified surface layer, the upper ocean of the Labrador Sea (the upper 300–500 m) was also cooler and fresher in OS2016. The large body of relatively fresh LSW was slightly cooler (−0.25 to −0.5 °C) and notably fresher in the deepest layer (−0.04 centered on 1,500 m), which, as we saw in the Irminger Sea, is presumably the signature of deeper winter convective mixing after OS2014. In the Labrador Sea we observe a net southward transport of LSW in OS2014 (−8.3 ± 9.3 Sv) and less in OS2016 (−1.8 ± 9.4 Sv) although the difference lies within our uncertainty range and is not significant.

The overflow layer is thicker in the deep Labrador Sea than anywhere else in the section, from around 2,000 to the seafloor at ~3,800 m. Here the property changes from OS2014 to OS2016 are positive but very small (<0.25 °C and <0.02 in salinity; Figure 6). The circulation is cyclonic, and, as expected, the layer had near‐zero net transport in both years (1.4 ± 7.1 Sv in OS2014 and 0.5 ± 8.0 Sv in OS2016; Figures 4 and 5).

## 5 MOC and Fluxes

**Figure 7:**
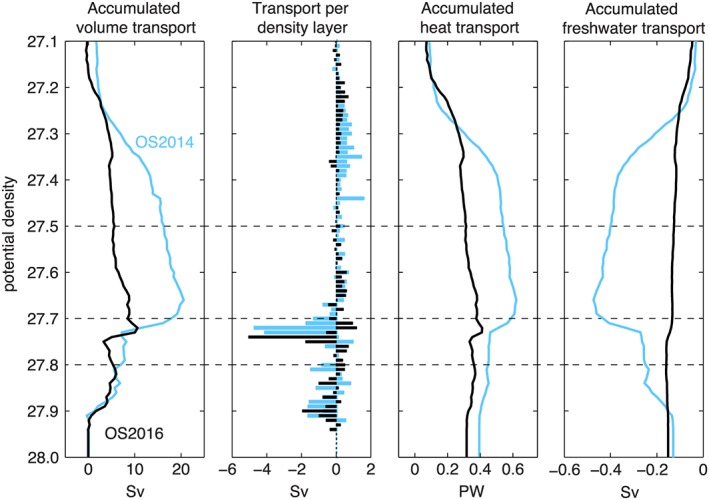
Volume and property transport profiles in potential density space for OS2014 (blue) and OS2016 (black; see section 3 for definitions and methods). Variables are accumulated from low to high density. Key isopycnals marked with dashed lines.

Profiles of transport integrated across the sections in density space are shown in Figure 7, with the accumulated profiles showing data from 27.10 to 28.00 kg/m^3^ (lighter water not shown). As expected, the majority of the northward transport is in the layer lighter than ~27.70 kg/m^3^, which contains the warm and saline NAC upper ocean and thermocline waters in the eastern basins. In OS2014 most of that northward transport is found in the density range 27.25–27.50 kg/m^3^, with transport maxima in layers associated with bodies of SPMW, for example, 27.45 kg/m^3^, which is the mode water east of the Irminger Current. In OS2016 the transport in the upper ocean is markedly reduced in total and shifted to slightly less dense layers (27.20–27.35 kg/m^3^) associated with the cooler and fresher NAC waters described earlier. The lightest layer (<27.1 kg/m^3^, not shown) includes some southward transport in both sections: This is the cold, fresh, Arctic‐origin waters of the Greenland and Labrador Shelf currents. The density range 27.50–27.70 kg/m^3^ (Figure 7) includes thermocline waters of the eastern basins (east of the Reykjanes Ridge), which are part of the NAC system (Figures 2 and 3). West of the ridge, however, this layer consists of the fresh and stratified near‐surface layers in the Irminger Sea and Labrador Sea and has a net southward transport.

Apart from a small southward transport in the very lightest waters, the northward transport is mainly balanced by southward transport below ~27.70 kg/m^3^ in the LSW and overflows. Between OS2014 and OS2016 the transport in the LSW switched from lighter to denser layers presumably associated with the deeper winter mixing observed in winter 2014/2015 in the Labrador and Irminger Seas. The total LSW transport was also reduced in OS2016. In contrast, the total transport in the overflow layers was markedly similar in both sections.

The reduced total transport in the warm, saline upper ocean of the eastern basins in OS2016 means that our estimates of net overturning circulation also have a large difference between the two sections (Table 3). The AMOC_*σ*‐max_ was 20.6 Sv ± 4.7 in OS2014 and 10.6 ± 4.3 Sv in OS2016. The AMOC_*σ*‐*n*_ estimates are higher than AMOC_*σ*‐max_; in OS2014 it was 23.3 ± 0.69 Sv and in OS2016 it was 13.0 ± 0.67 Sv. We discuss the meaning and relevance of the AMOC_*σ*‐*n*_ in section 7.

**Table 3:** Estimates of Overturning Circulation, Isopycnal Circulation and Heat and Freshwater Transport With Uncertainties (See Section 3)

Parameter	OS2014	OS2016
AMOC_*σ*‐max_	20.6 ± 4.7 Sv	10.6 ± 4.3 Sv
AMOC_*σ*‐*n*_	23.3 ± 4.7 Sv	13.0 ± 4.3 Sv
Maximum isopycnal transport	−41.4 ± 8.2 Sv	−58.6 ± 7.4 Sv
Total heat flux (HT)	0.39 ± 0.08 PW	0.32 ± 0.13 PW
Isopycnal heat transport (HT_gyre_)	0.17 ± 0.02 PW	0.21 ± 0.02 PW
Total freshwater flux at section (FT)	−0.21 ± 0.03 Sv	−0.25 ± 0.08 Sv
Isopycnal freshwater transport (FT_gyre_)	−0.10 ± 0.02 Sv	−0.16 ± 0.03 Sv

The total heat and freshwater transport profiles in density space are also shown in Figure 7; these profiles are the heat and freshwater transport in density bands accumulated from the lightest to most dense layers. The net property transport or flux at the OSNAP section is the value reached at the deepest layer; in OS2014 the heat and freshwater fluxes were 0.39 ± 0.08 PW and −0.21 ± 0.03 Sv, while in OS2016 they were 0.32 ± 0.13 PW and −0.25 ± 0.08 Sv, respectively. It is clear that the two sets of property transport profiles have different vertical structures that reflect the differences in the transport profiles, and in the case of the OS2016 freshwater transport, the salinity distribution, as follows. The upper layer in OS2016 has less northward transport of volume and heat and less upper layer southward freshwater transport (Figure 7). The smaller overall heat transport in OS2016 leads intuitively from the smaller overturning circulation; however, the freshwater transport has a different vertical structure, with changes in both the upper and deep layers. In OS2014 there was more southward transport of freshwater in the upper layer (<27.7 kg/m^3^) and more northward freshwater transport in the deep layer (>27.7 kg/m^3^, right‐hand panel Figure 7), though the net transport was smaller than OS2016. In the next section we examine the distribution of volume, heat, and freshwater transport against distance along the section in order to define the contribution of the gyre‐scale circulation to property transport.

## 6 Isopycnal Circulation and Fluxes

**Figure 8:**
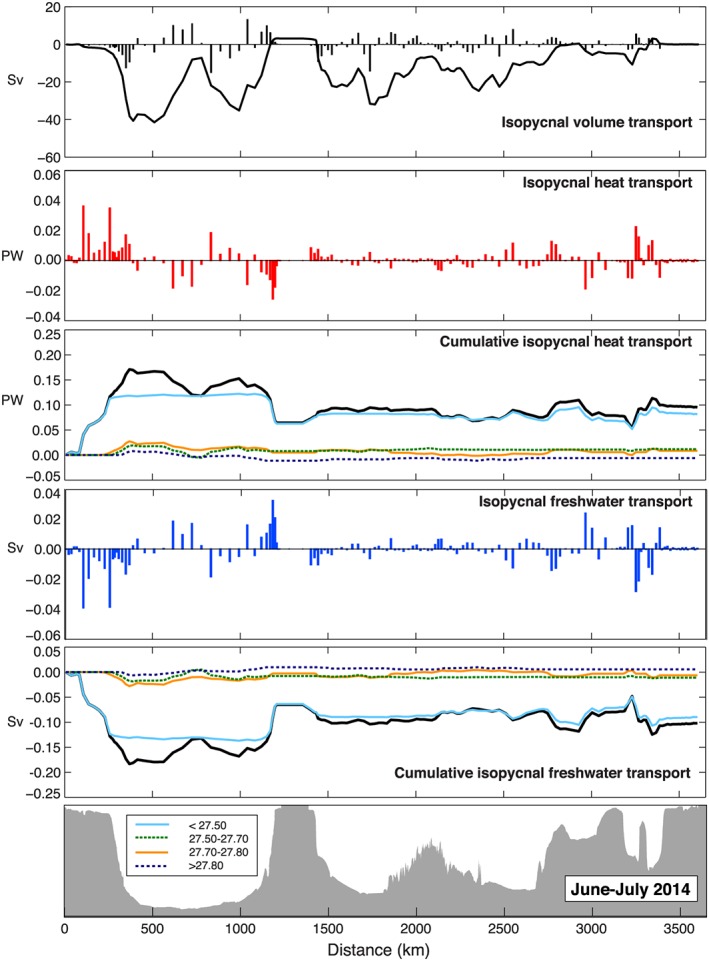
Along‐section profiles of isopycnal volume and property transport from OS2014 (see section 3 for definitions and methods). Bars indicate total isopycnal transport at each station pair along the track. Curves represent transport accumulated from west to east; solid black lines are surface to seafloor total, and colored lines indicated transport in potential density ranges (color scale given in bottom panel).

**Figure 9:**
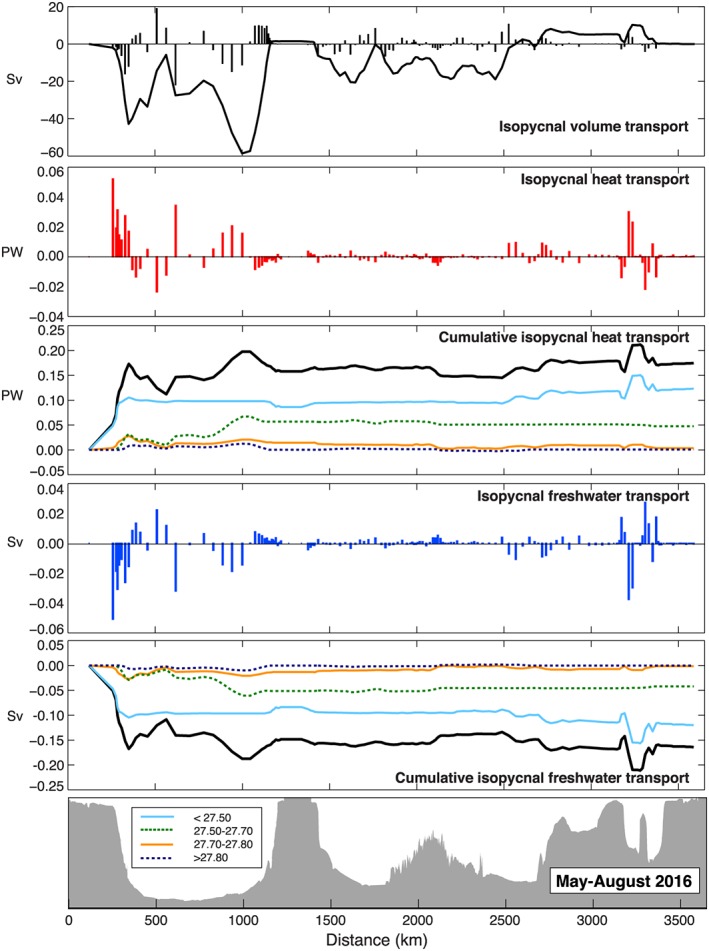
Along‐section profiles of isopycnal volume and property transport from OS2016 (see section 3 for definitions and methods). Bars indicate total isopycnal transport at each station pair along the track. Curves represent transport accumulated from west to east; solid black lines are surface to seafloor total, and colored lines indicated transport in potential density ranges (color scale given in bottom panel).

In section 3, we described how the velocity and transport fields can be decomposed into throughflow, overturning (diapycnal), and gyre‐scale (isopycnal) circulation. By definition both the overturning and isopycnal circulation sum to zero transport, but their associated heat and freshwater transport components do not because of the temperature and salinity gradients. We find that the isopycnal transport (the maximum in *T*_gyre_ accumulated from west to east) was −41.4 ± 8.2 Sv in OS2014 and −58.6 ± 7.4 Sv in OS2016 (Table 2 and Figures 8 and 9). In both sections the maximum was located in the mid‐Labrador Sea. The isopycnal heat and freshwater transport estimates are 0.17 ± 0.03 PW and −0.10 ± 0.02 Sv for OS2014 and 0.21 ± 0.03 PW and −0.16 ± 0.03 Sv for OS2016 (Table 2).

The west‐east profiles of the isopycnal volume, heat, and freshwater transport (Figures 8 and 9) show that the boundary currents especially in the Labrador Sea and the eastern Iceland Basin, Hatton‐Rockall Basin, and the Rockall Trough are where the isopycnal property transport is highest. This finding highlights the need for observations in those locations: For example, we note that the OS2014 section has more stations close to the coast at the western end of the section and that a large amount of freshwater transport was observed midshelf. These stations were not sampled in OS2016, and it is possible the net freshwater transport for that section is underestimated as a result. It also highlights the importance of variability in the eastern basins to the net property transports; the biggest difference between OS2014 and OS2016 isopycnal and diapycnal heat and freshwater transport is found in the warmest and most saline upper waters of the eastern basins (the blue lines in Figures 8 and 9) where mesoscale and temporal variability has been observed to be highest (Holliday et al., 2015; Zhao et al., 2018).

Finally, we note that for OS2014 almost all of the isopycnal property transport was found in the upper layer (<27.50 kg/m^3^), while for OS2016 after deep winter convection east of Greenland occurred, the intermediate (thermocline/SAIW) layer also carried significant isopycnal heat and freshwater transport (Figures 8 and 9). It appears to be this extra heat and freshwater transport that leads to the significantly increased total isopycnal heat and freshwater transport in OS2016.

## 7 Discussion

In this section we place our results into context by comparing them with findings from the literature, and we discuss uncertainties in our estimates that are in addition to our quantified methodological uncertainties.

From two OSNAP hydrographic sections we have described the details of the velocity, density, temperature, and salinity fields. We report estimates of the overturning circulation (AMOC_*σ*‐max_) at the time of the sections that span a large range: 20.6 ± 4.7 Sv for OS2014, and 10.6 ± 4.3 Sv for OS2016. To our knowledge there are no existing estimates of overturning circulation from an equivalent section that includes the Labrador Sea and the eastern subpolar North Atlantic. However, our AMOC estimates are similar (within error bounds) to the wide range of estimates reported from the OVIDE section (Greenland to Portugal). That line has been repeated several times with similar measurements to the OSNAP hydrographic section and provides a mean estimate of the subpolar AMOC_*σ*_ of 16.0 Sv with a range of 11.4–18.5 Sv (Mercier et al., 2015). In the same analysis (Mercier et al., 2015), altimeter‐based estimates of AMOC_*σ*_ suggest a range of less than 15 to more than 25 Sv. The 2014 occupation of the OVIDE section, taken very close in time to the OS2014 section, gives an AMOC_*σ*_ of 18.7 ± 3.0 Sv (Zunino et al., 2017), slightly less than our estimate that includes the Labrador Sea. The 2014 OVIDE estimate is closer to the findings of Rossby et al. (2017) that report an AMOC_*σ*_ of 18.3 ± 3.4 Sv from repeated ADCP measurements at 60°N between Greenland and Scotland, and Sarafanov et al. (2012) that report an AMOC of 16.5 ± 2.2 Sv from repeated hydrographic sections and altimetry also at 60°N east of Greenland.

A second way to view diapycnal circulation is using AMOC_*σ*‐*n*_, which is the sum of all the northward transport in the upper layer (lighter than the density at the maximum value of the overturning stream function). AMOC_*σ*‐*n*_ estimates are higher than AMOC_*σ*‐max_ because in the latter the net transport of the upper limb includes (is reduced by) the southward transport of very light and shallow waters of the EGC and Labrador Current systems. AMOC_*σ*‐*n*_ represents the total volume of northward flowing warm saline upper ocean water that is diapycnally transformed both into a returning (southward flowing) cold, denser layer and into a returning (southward flowing) cold, fresher, and lighter layer. The lighter, fresher return flow layer has been transformed in the Arctic region by mixing, air‐sea fluxes, and melt water from land ice and sea ice. In contrast, the AMOC_*σ*‐max_ only represents the volume of warm, saline upper layer that through diapycnal transformation becomes a returning cold dense layer.

The OS2016 exhibits features that reflect the occurrence of deep convective mixing in the Labrador Sea and the Irminger Sea in the winters following OS2014 (deep mixed layers, with cooling and freshening in the deep LSW recently ventilated). We note, however, that despite the increased production of LSW in the winters of 2014/2015 and 2015/2016, the net export of the LSW layer from the full‐width Labrador Sea in 2016 appeared to be less during OS2016 (−1.8 ± 9.4 Sv) than during OS2014 (−8.3 ± 9.3 Sv), although the difference is not significant because of the large uncertainty range.

There are two surprising aspects to the isopycnal heat and freshwater transport estimates; the first is that the isopycnal property transports are up to 65% of the total property transport. This is higher than then 10% and 45% for horizontal heat and freshwater transport observed at 26°N and computed in depth space (McCarthy et al., 2015; McDonagh et al., 2015). It is also contrasts with Mercier et al. (2015) who find that the isopycnal heat transport is typically 10% of the total at the OVIDE section. Figures 8 and 9 show that the largest isopycnal heat and freshwater transport is found in the west Labrador Sea, not sampled by OVIDE, and this is likely the reason for the difference between these results and those of Mercier et al. (2015). The second surprising aspect is the large range in these values from the two OSNAP sections, with higher isopycnal heat and freshwater transport observed in OS2016 when the overturning circulation was lower.

The AMOC estimate derived from OS2016 is lower than OS2014 but within the range observed by the OVIDE program. However, there are questions about additional uncertainty associated with a section made up of expeditions collecting data over a 3‐month period. We consider two possible sources of additional uncertainty: mismatches in the density field where the cruise data sets join and the seasonal cycle in properties and stratification, as follows. The OS2016 section is composed of data from four cruises (Table 1), with the boundaries located at Greenland (i.e., land), the Reykjanes Ridge (1,100‐m water depth), and Rockall (100‐m water depth). Potentially erroneous extra transport could result from a significant change in the density structure in the time between the two stations. The choice of Greenland and Rockall Island as two boundary points excludes the possibility of a spurious density gradient at those locations. At the Reykjanes Ridge the two 2016 stations were taken 6 weeks apart, and there was very little change in density structure of those and their immediate neighbor stations, and close to zero transport was observed between them in OS2016 and between the equivalent stations in OS2014 (Figures 4 and 5). All four cruises took place in the summer months of May–August, but there exists the potential for a seasonal cycle in circulation and properties to cause some uncertainty in our results. Information on the seasonal variability in circulation and property transport in this region is sparse because most measurements take place in spring‐summer. Rossby et al. (2017) find no significant changes in the transport in the May–August period in all the major currents between Greenland and Scotland, except the EGC, which has higher transport in May. Mercier et al. (2015) find from altimetry data that while the AMOC_*σ*_ has a seasonal cycle, the annual minimum takes place during the months of May to August. Gary et al. (2018) show that an expected wind‐driven seasonal cycle in transport in the eastern basins is not detectable above the high mesoscale and submesoscale variability there. From these results we conclude that there is no evidence to suggest that the use of OS2016 sampled between May and August introduced an unreasonable uncertainty due to appending four cruises, or to undersampling the seasonal cycle in transport.

As far as we are aware there have been no previous observation‐based estimates of the role of the gyre‐scale isopycnal circulation in the transport of heat and freshwater through the full width of the subpolar North Atlantic, including the Labrador Sea. Our finding that isopycnal heat and freshwater transport is high is significantly different from the negligible isopycnal property transport found at this section in a high‐resolution ocean model (Xu et al., 2016) and at the OVIDE section (Mercier et al., 2015). Similar to our wide range of overturning circulation estimates, we note a wide range of isopycnal circulation from our two sections. While we observe that higher heat flux during OS2014 is associated with higher overturning transport and higher freshwater flux during OS2016 is associated with the higher isopycnal transport (Table 3), we cannot say with any certainty whether those relationships persist over other time periods.

A key finding is that the magnitude of property transport by the isopycnal circulation is sensitive to the geographic extent of the observations, because the highest property fluxes are found in the narrow boundary currents of the Labrador Sea and the basins east of the mid‐Iceland Basin. There is some debate in the literature as to whether the eastern boundary currents are part of the subpolar gyre or not. In basin‐integrated studies the entire region is often called the subpolar gyre, but other studies seek a boundary of the gyre in order to investigate changes in gyre dynamics. The eastern subpolar gyre boundary is often defined as a density/salinity front in the east Iceland Basin (the Subpolar Front, e.g., Bersch et al., 2007; Lozier & Stewart, 2008; Zunino et al., 2017), or recently by closed contours of sea surface height (Foukal & Lozier, 2017). The latter definition excludes the shallowest parts of the western boundary currents and all of the eastern basins, that is, the parts of the isopycnal circulation where our results show that most of the property transport takes place. Our definition of isopycnal circulation includes the central gyre and also allows for a wider regional circulation of the warm, saline eastern waters and returning fresher and cooler western waters.

Our two sections add further synoptic views of the transport in the principal circulation features of the region, which we next compare to the literature. The comparison serves the purpose of understanding the context and representativeness of our sections and estimates, without attempting to infer any insight into change over time. The transport in the Rockall Trough has a very high range, which hints at the difficulty of measuring transport in a region of energetic mesoscale and submesoscale recirculation. Although the range of our two estimates of transport in the upper layer is high (<27.50 kg m^3^, −0.7 ± 0.9 Sv, and 7.6 ± 1.0 Sv; Table 2), they lie within the range estimated from four decades of historical temperature and salinity data in the same location (Holliday et al., 2015, 2000). A study of direct observations from SADCPs (Rossby et al., 2017) shows a similar wide range in directly measured velocity north of the Rockall Trough, which the authors suggest may be related to circulation around seamounts. We cannot yet explain the large range of transports in this eastern basin, and further insight will come from the continuous records from the OSNAP moorings located here. However, our results do show that this is also a key region for heat and freshwater transport because they are very warm and salty, and since transport dominates those terms, it is important that we resolve these variations adequately.

In the Iceland Basin the two NAC jets are unusual in their consistency of location and transport in the two sections (6.9–7.9 and 7.3–7.5 Sv, combined upper and thermocline layers <27.70, see Table 2). The NAC total of 14.2–15.4 ± 4.8 Sv is very close to an estimate of 15.0 ± 0.8 Sv at 60°N (Sarafanov et al., 2012) and close to the range of the means for these two NAC branches from OVIDE NAC (11.4 ± 5.1 Sv, Daniault et al. (2016)).

The upper ocean and thermocline of the Iceland Basin, Hatton‐Rockall Basin, and the Rockall Trough all show notable cooling and freshening between the sections, with largest changes evident in the NAC jets. The source of the freshening is an ongoing research topic, and we make no attempt to explain it here: Instead, we note that there has been evidence of a decadal‐scale decline in eastern subpolar North Atlantic salinity since ~2008 (e.g., Holliday et al., 2015), which may be related to long‐term changes in freshwater transport convergence by the overturning circulation (e.g., Robson et al., 2016), potentially reinforced by shorter term air‐sea flux anomalies (e.g., Zunino et al., 2017), or related to gyre changes through mechanisms described by Hátún et al. (2005). Meanwhile, we note that in contrast to the fresher eastern basins in OS2016, the Greenland boundary currents (the inshore EGC/EGCC and the inshore WGC) are warmer and more saline in OS2016 than in OS2014. It is not clear whether this is a consequence of undersampling of high‐frequency variability or represents a longer‐term trend.

The boundary currents of the Irminger and Labrador Seas have deep reaching current systems with a strong barotropic component. Our section estimates lie within the range of other similar hydrography‐based estimates, as follows. In the Irminger Sea the full‐depth western boundary current system transported −23.5 ± 3.2 to −27.0 ± 2.7 Sv, within the literature estimate range of −23.7 to −40.5 Sv (Daniault et al., 2016; Holliday et al., 2009; Mercier et al., 2015; Sarafanov et al., 2012). In the Labrador Sea the western boundary current system transported −30.8 ± 2.5 to −41.9 ± 1.8 Sv, while literature estimates range from −56 Sv for the full gyre (Hall et al., 2013) to −30.2 ± 6.6 Sv (>400 m, Zantopp et al., 2017). However, we offer two caveats for our new estimates. The first is to reinforce the point made earlier that the total transport in a current system with a boundary in mid‐ocean is highly dependent on the choice of location of the boundary, which can be obscured by the presence of eddies or recirculation features. The second is that the method of data collection (sequential stations over a number of days) undersamples high‐frequency variability with the boundary currents such as variability introduced by topographic waves (Fischer et al., 2015; Zantopp et al., 2017).

Finally, we consider transport in the overflow layers and how these compare to literature estimates. In the OS2014 and OS2016 sections, the velocity in the dense overflow layer is rather different compared to the overlying LSW, with narrow currents that are probably highly turbulent (e.g., Lauderdale et al., 2008) and that do not seem well resolved in either space or time by our sections. Along with the issue that measures of transport within these two deep layers are sensitive to the horizontal and vertical (density‐based) boundary definitions, our estimates clearly have some uncertainty. However, across the sections they describe a coherent picture of a gradually increasing volume of overflow waters (>27.80 kg/m^3^) as they circulate cyclonically from the sills to the Labrador Sea. In the Iceland Basin the net overflow transport in OS2014 was −2.9 ± 2.7 Sv of ISOW (though only 0.1 ± 2.4 Sv in OS2016), which is within the range of previous estimates of −2.1 to −3.9 Sv (Daniault et al., 2016; Holliday et al., 2015; Kanzow & Zenk, 2014; Sarafanov et al., 2012). Our estimates of the overflow layer in the Irminger Sea western boundary current (which includes modified ISOW and DSOW) of −6.9 ± 0.9 to −8.7 ± 1.1 Sv are on the low side compared to equivalent literature estimates of −9.0 to −12.3 Sv (Bacon & Saunders, 2010; Holliday et al., 2009; Lherminier et al., 2010; Sarafanov et al., 2012). However, our estimates of the western Labrador Sea overflow layer (ISOW and DSOW) of −12.5 ± 1.0 to −15.1 ± 0.5 Sv are consistent with the long‐term mean transport in the overflow layer at the 53°N array (−15.7 ± 2.7 Sv, Zantopp et al., 2017).

## 8 Summary

Two highly spatially resolved CTD/LADCP sections have been analyzed to estimate the total full‐depth velocity field across the subpolar North Atlantic between Canada, Greenland, and Scotland. The velocity fields show the expected cyclonic gyre‐scale upper layer circulation and additionally provide accurate new insight into transport and circulation within the intermediate and deep layers. We have computed volume transport and decomposed it into the throughflow, overturning circulation, and gyre‐scale isopycnal circulation and estimated the associated components of heat and freshwater transport.

The two sections show a wide range in the estimates of the overturning circulation: the AMOC_*σ*_‐_max_ in OS2014 was 20.6 ± 4.7 Sv and in OS2016 was 10.6 ± 4.3 Sv. For both sections the AMOC_*σ*_‐_*n*_ values were ~3 Sv higher: 23.3 ± 4.7 Sv in OS2014 and 13.0 ± 4.3 Sv in OS2016. We have found that the strength of the overturning circulation is not an indicator of the strength of the gyre‐scale isopycnal circulation; during our two sections the isopycnal circulation was stronger when the overturning was weaker (−41.4 ± 8.2 Sv in OS2014 and −58.6 ± 7.4 Sv in OS2016).

The total heat and freshwater fluxes were 0.39 ± 0.08 PW and −0.21 ± 0.03 Sv in OS2014 and 0.32 ± 0.13 PW and −0.25 ± 0.08 Sv in OS2016. Thus, heat flux was higher when the MOC was largest, but freshwater flux was greater when the isopycnal circulation was increased. The isopycnal components of heat and freshwater transport were major contributors to the total flux: up to 65%, and the majority of the heat and freshwater transport was found in the western Labrador Sea (where water is very cold and fresh) and the eastern basins (east Iceland Basin, Rockall‐Hatton Plateau, and Rockall Trough, where water is warm and salty).

The upper layer property fields changed between the two sections, with notably cooler and fresher conditions in Iceland Basin and Rockall Trough in OS2016. The deepest layers of the Labrador Sea and Irminger Sea exhibited cooling and freshening after deep winter convection after OS2014; interestingly the development of a thicker layer of ventilated LSW did not result in higher export of LSW from the Labrador Sea in OS2016. However, there was more isopycnal transport of freshwater and heat within the intermediate layer in OS2016.

The estimates of transports within major currents in our two sections are within the range of observations from the literature. Uniquely, however, these two sections provide the first highly spatially resolved observations of the total velocity field in sections that traverse both the Labrador Sea and the eastern subpolar North Atlantic.
